# Genome-wide association analysis reveals 6 copy number variations associated with the number of cervical vertebrae in Pekin ducks

**DOI:** 10.3389/fcell.2022.1041088

**Published:** 2022-11-10

**Authors:** Yaxi Xu, Jian Hu, Wenlei Fan, Hehe Liu, Yunsheng Zhang, Zhanbao Guo, Wei Huang, Xiaolin Liu, Shuisheng Hou

**Affiliations:** ^1^ Key Laboratory of Animal (Poultry) Genetics Breeding and Reproduction, Ministry of Agriculture; Institute of Animal Science, Chinese Academy of Agricultural Sciences, Beijing, China; ^2^ College of Animal Science and Technology, Northwest A&F University, Yangling, Shanxi, China

**Keywords:** number of vertebrae, cervical vertebra, duck, copy number variation, GWAS

## Abstract

As a critical developmental stage in vertebrates, the vertebral column formation process is under strict control; however, we observed variations in the number of cervical vertebrae in duck populations in our previous study. Here, we further explored the variations in the number of vertebrae in two duck populations: 421 Pekin duck × mallard F2 ducks and 850 Pekin ducks. Using resequencing data of 125 Pekin ducks with different numbers of cervical vertebrae and 352 Pekin duck × mallard F2 ducks with different numbers of thoracic vertebrae, we detected whole-genome copy number variations (CNVs) and implemented a genome-wide association study (GWAS) to identify the genetic variants related to the traits. The findings verified the existence of variations in the number of cervical vertebrae in duck populations. The number of cervical vertebrae in most ducks was 15, while that in a small number of the ducks was 14 or 16. The number of cervical vertebrae had a positive influence on the neck production, and one cervical vertebra addition could increase 11 g or 2 cm of duck neck. Genome-wide CNV association analysis identified six CNVs associated with the number of cervical vertebrae, and the associated CNV regions covered 15 genes which included *WNT10A* and *WNT6*. These findings improve our understanding of the variations in the number of vertebrae in ducks and lay a foundation for future duck breeding.

## Introduction

Animals’ spinal column has important functions, such as supporting the trunk, protecting the internal organs and the spinal cord, and the formation process of the vertebral column is under strict control. While variations in the number of vertebrae have been widely observed in livestock populations, which could increase the body size of animals and improve meat production (Huang et al., 2017; Zhang et al., 2017), studies on the variations in the number of vertebrae were first conducted in pig populations and has currently made the most visible progress in pig populations (Mikawa et al., 2005). From 1998 to the present day, using the backcross resource family (Rohrer and Keele, 1998), the F2-generation resource population (Sato et al., 2003; Fan et al., 2013), and the F3-generation population (Wada et al., 2000; Borchers et al., 2004), two QTLs (quantitative trait loci) affecting the spine number variations of pigs have been located, which provide molecular markers for the selection for improving the body size of Chinese local pig breeds (Mikawa et al., 2011; Fan et al., 2013). Furthermore, variations in the number of vertebrae have also been observed in yak, cattle, and sheep populations, and several candidate genes have been found (Han et al., 2013; Li et al., 2019; Wang et al., 2020).

In response to the variations in the number of vertebrae in birds, as early as 1868, Darwin had discovered variations in the number of cervical vertebrae in mallard populations (Darwin, 1868). After that, variations in the number of vertebrae in birds had been rarely reported.

Ducks are an important resource of poultry meat and have a large market demand. In China, a country with a splendid catering culture, ducks are popular not only for their breast meat or leg meat but also for their neck, head, tongue, wings, feet, liver, and so on. Among these, the duck neck, as a kind of leisure food, is widely and well received by consumers. Therefore, it may be of certain economic worth in breeding duck populations with a high neck production. However, it is not easy to measure neck production *in vivo*. The breeding for high neck production might need to rely on the molecular breeding method.

The copy number variation (CNV) is a kind of genomic variation which could affect gene expression and consequently the phenotypes by changes to the gene structure and dosage (Zhou et al., 2011). In human and mouse studies, the CNV was found to account for 18%–30% of the genetic variation in gene expression and showed critical roles in both phenotypic variability and disease susceptibility (Stranger et al., 2007; Henrichsen et al., 2009). In domestic animals, CNV has been widely studied in cattle (Gao et al., 2017; Mielczarek et al., 2017), chicken (Zhang et al., 2014), pig (Zhou et al., 2016), sheep (Ma et al., 2017), goats (Fontanesi et al., 2010), horse (Kader et al., 2016), and so on. However, no study has been conducted in duck populations.

Here, we explored the variations in the number of cervical and thoracic vertebrae in duck populations. Combining the phenotypes with whole-genome sequence data, we aimed to detect the genetic variants affecting the phenotypic variation, which would improve our understanding of the genetic mechanisms underlying the variations in the number of vertebrae in ducks and lay a foundation for future duck breeding.

## Materials and methods

### Animals

The populations involved in this study included a Pekin duck × mallard F2 intercross population (Zhou et al., 2018), established in the Institute of Animal Science, Chinese Academy of Agricultural Sciences, and a Pekin duck population from Sai Fei Ya company in China.

### Phenotype collection and sampling

All the phenotypes were collected after slaughter. A total of 421 ducks from the F2 population were slaughtered at 49 weeks of age. The number of cervical vertebrae (CVN), neck weight, neck length, neck weight percentage (neck weight/carcass weight), number of thoracic vertebrate (TVN), keel length, breast meat weight, and breast meat percentage (breast meat weight/carcass weight) were measured. The cervical and thoracic vertebral morphometry was predicted using X-ray photography and spine specimen preparation.

The experiment on the Pekin duck population was conducted in one slaughterhouse of the Sai Fei Ya Company where 40 million ducks are slaughtered in 1 day. All the Pekin ducks were slaughtered when they were 6 weeks of age. 850 ducks were randomly selected to count the number of cervical vertebrae and detect the distribution of ducks with different phenotypes in this Pekin duck population. Meat samples of 125 Pekin ducks with different phenotypes were taken for DNA extraction for resequencing. The phenotypes of the 125 Pekin ducks for genome-wide CNV analysis are shown in Table 3.

### Whole-genome resequencing

Total genomic DNA was extracted with a traditional phenol–chloroform method. The quality and quantity of the 125 DNA samples were examined with a Nanodrop 2000 system (Thermo Fisher Scientific, Illkirch, France) and by agarose gel electrophoresis. Then, paired-end libraries were generated for the eligible samples using standard procedures with an average insert size of 500 bp and an average read length of 150 bp. All libraries were sequenced on an Illumina Hiseq X-Ten (Illumina, San Diego, United States) platform with an average raw read sequence coverage of 6× for the 125 Pekin ducks. Whole-genome sequences of 352 Pekin duck × mallard F2 ducks (consisting of 170 ducks with 9 thoracic vertebrae and 182 ducks with 10 thoracic vertebrae) were retrieved from our previous study (Liu et al., 2021).

### Copy number variation segmentation and genotyping

The raw reads were mapped to the duck reference genome *duckbase.refseq.v4* (https://www.ncbi.nlm.nih.gov/assembly/GCA_900411745.1) with Burrows–Wheeler Aligner (BWA aln) v 0.7.8 (Li and Durbin, 2009) with default parameters. On average, 84.47% of the reads were mapped, resulting in a final average sequencing coverage of 7.2× (ranging from 6× to 10×) per individual.

Using the BAM files generated by the sequence alignment of all individuals, the GC content, repeat and gap content, number of reads, and the absolute copy number of each window were calculated using a slide-window approach (1 kb sliding windows with 200 bp steps) with the CNVcaller program (Wang et al., 2017). According to the size of the experimental population, the genome-wide CNV regions and genotypes were determined with the correlation of r of 0.2 and 0.15 for the Pekin duck population and the F2 population, respectively.

The package lumpy-sv was then used to search for possible structure variants in the genome based on the breakpoint sequence, sequencing depth, and other prior knowledge in a probability box. Using the bam files generated by the sequence alignment of all individuals, discordants and splitters were compiled with the program SAMtools v1.0 (Li, 2011). Based on the original BAM file and the two newly generated BAM files, the structural variation detection was carried out using the Lumpy program (Layer et al., 2014).

The CNV regions (CNVRs) detected at the genome-wide scale were filtered using conditions with boundary factors greater than 0.5 and lengths greater than 4 kb.

### Statistical analyses

In this study, general statistical analyses were performed using the SAS v9.4 program. The ANOVA test was performed to compare the difference.

Using the filtered genome-wide CNVRs, a genome-wide association study was performed on the number of cervical vertebrae with a general linear model (GLM) in TASSEL (Bradbury et al., 2007). The analysis model is as follows:

p=mean+cnv effect+sex+pcs+e

The population structure (pcs) and sex effects (sex) are considered to minimize false positives and increase statistical power as much as possible.

The whole-genome significance cutoff was defined as the Bonferroni test threshold with 0.01/total variants or 0.05/total variants.

### Copy number variation validation by quantitative real-time polymerase chain reaction

Moreover, the six CNVRs associated with the number of cervical vertebrae were validated by quantitative real-time PCR (qPCR). Primers were designed in the CNVRs using the software Primer Premier 5 (Supplementary Table S1). Following a previous study, the *Ldh-B* gene served as the reference gene (Hendriks et al., 1988). The qPCR experiments were conducted using the QuantStudio™ 6 Flex Real-Time PCR Systems (Life Technologies, Carlsbad, CA, United States). The qPCR was performed in a reaction volume of 25 μl, consisting of 12.5 μl of 2× SYBR Green qPCR Mix (Life Technologies, Carlsbad, CA, United States), 1 μl (10 pmol/μL) of each primer (forward and reverse), 2 μl of template DNA (30 ng/μl), and 8.5 μl of ddH_2_O. The thermocycling condition included an initial denaturation at 95°C for 10 min, 40 cycles for the next three steps, such as denaturation at 95°C for 15 s, annealing at 60°C for 15 s, and extension at 72°C for 1 min, and then a final extension at 72°C for 10 min. For qPCR, the relative copy numbers were estimated by the 2^−ΔΔCt^ method. The value of 2×2^ΔΔ*C*T^ between 1.5 and 3 was considered most likely to represent a normal copy number of 2, and below 1.5 or above 3 was considered deletions or duplications, respectively.

## Results

### Phenotypic variations of cervical and thoracic vertebrae

**FIGURE 1:**
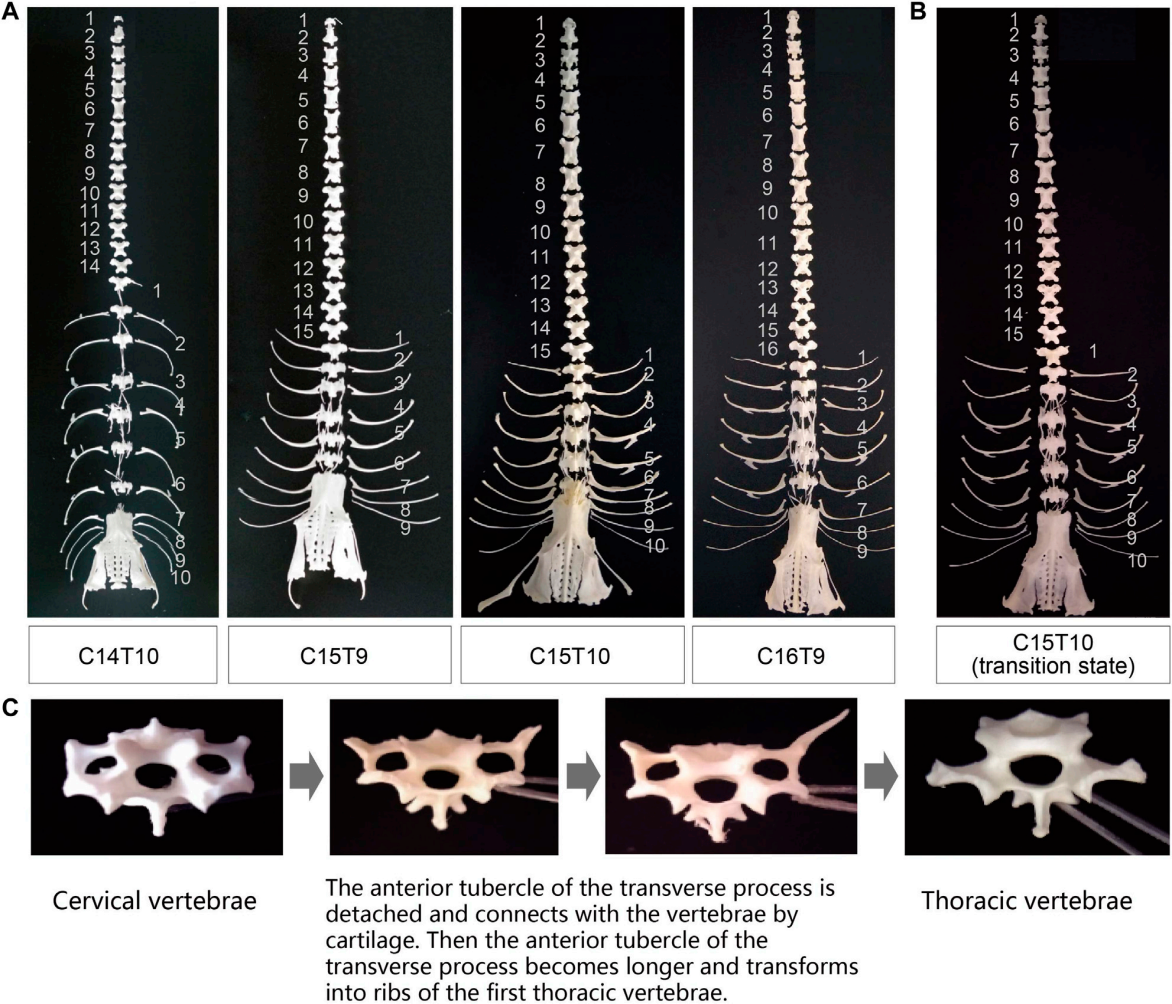
Phenotypic variation in the number of cervical and thoracic vertebrae in ducks. **(A)** Specimens of duck axial skeletons with C14T10, C15T9, C15T10, and C16T9. C means the number of cervical vertebrae; T means the number of thoracic vertebrae. **(B)** Specimens and X-ray photography of the duck axial skeleton with C15T10 (15 cervical vertebrae and 10 thoracic vertebrae) in the transitional state with extremely short first ribs. **(C)** Morphological transition from the last cervical vertebra to the first thoracic vertebra.

**FIGURE 2:**
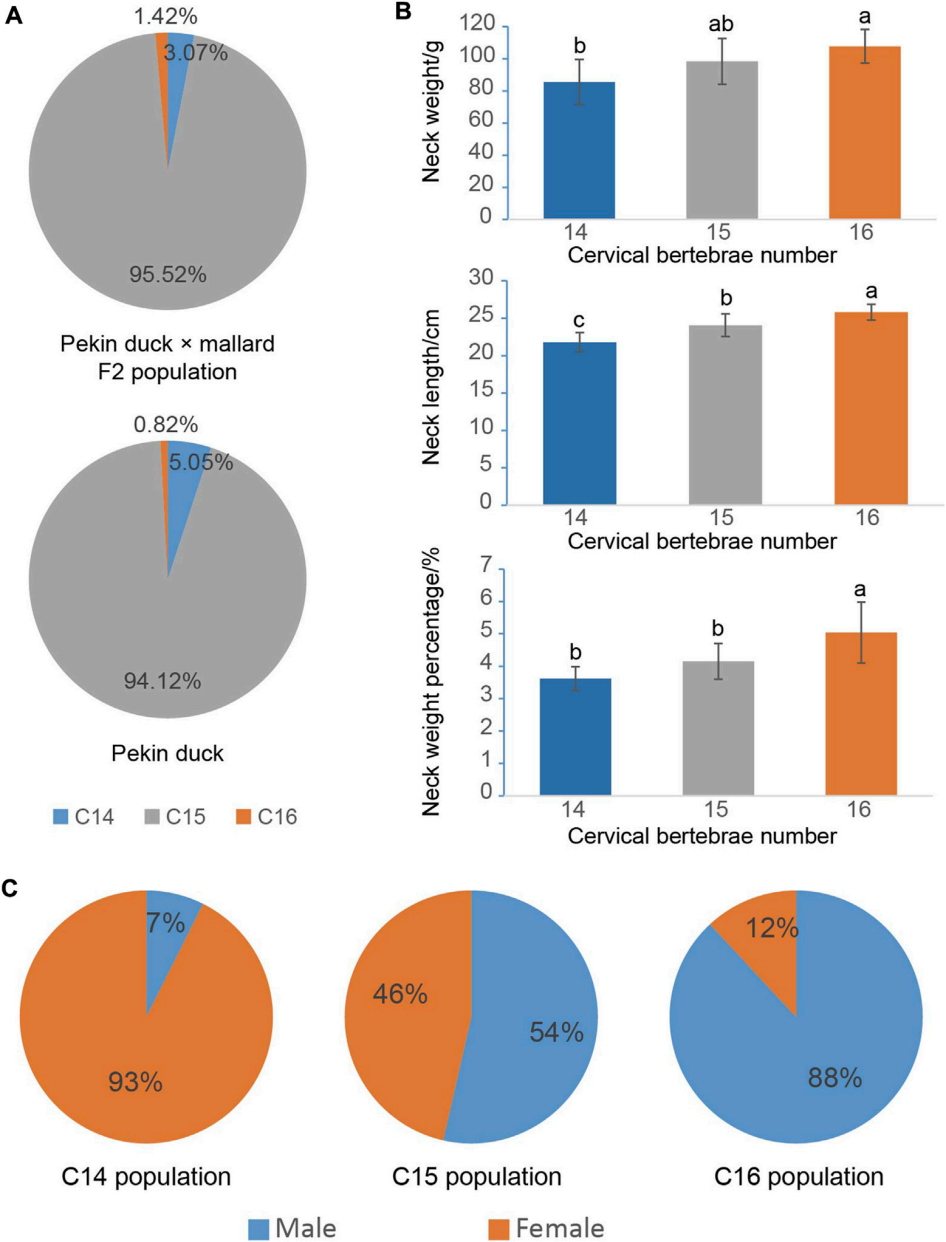
Phenotypic distribution of the cervical vertebra number and their effects on economic traits. **(A)** Phenotypic distribution of the cervical vertebra number in the Pekin duck × mallard F2 population and the Pekin duck population. **(B)** The effects of the cervical vertebra number on neck weight and neck length; alphabet differences between the groups represent statistically significant difference with the Duncan test; bars represent the standard deviation of the mean. **(C)** Distributions of C14 (14 cervical vertebrae), C15 (15 cervical vertebrae), and C16 (16 cervical vertebrae) phenotypes in the male and female Pekin duck population.

As presented in Figure 1A and Supplementary Figure S1, the F2 ducks exhibited cervical (C) and thoracic (T) vertebrae of C14T10, C15T9, C15T10, and C16T9. As seen in Supplementary Figure S1, the number of vertebrae could not be always clearly obtained from the X-ray photographs for the overlapped shadows. The phenotypic distribution is shown in Tables 1–3. Among the 421 F2 ducks, the percentage of ducks with C14T10, C15T9, C15T10, and C16T9 phenotypes were 3.09%, 43.47%, 52.02%, and 1.43%, respectively (Table 1). In the F2 duck population, the ducks with 15 cervical vertebrae made up the majority of the population (95.52%), while the ducks with 14 and 16 cervical vertebrae accounted for 3.07% and 1.42% of the population, respectively (Table 1; Figure 2A). 55% of F2 ducks had 9 thoracic vertebrae and the other 45% had 10 thoracic vertebrae (Table 1). As shown in Table 2, the C14, C15, and C16 ducks were also observed in the Pekin duck population. In randomly sampled 850 Pekin ducks, the C14, C15, and C16 ducks occupied 5.05%, 94.12%, and 0.82% of the population (Table 2; Figure 2A), respectively.

**TABLE 1:** CVN (number of cervical vertebrae) and TVN (number of thoracic vertebrae) distribution in the Pekin duck × mallard F2 population.

	Type	Male	Percentage (%)	Female	Percentage (%)	Sum	Percentage (%)
Cervical vertebrae	C14	0	0.00	13	4.05	13	3.09
	C15	95	95.00	307	95.64	402	95.49
	C16	5	5.00	1	0.31	6	1.42
Thoracic vertebrae	T9	33	33.00	156	48.60	189	44.89
	T10	67	67.00	165	51.40	232	55.11
Cervical and thoracic vertebrae	C14T10	0	0.00	13	4.05	13	3.09
	C15T9	28	28.00	155	48.29	183	43.47
	C15T10	67	67.00	152	47.35	219	52.02
	C16T9	5	5.00	1	0.31	6	1.42

**TABLE 2:** CVN (number of cervical vertebrae) distribution in the Pekin duck population.

Number of cervical vertebrae	N	Percentage (%)
14	43	5.05
15	800	94.12
16	7	0.82
Sum	850	100.00

**TABLE 3:** The distribution of ducks with different numbers of cervical vertebrae in the Pekin duck population used for genome-wide CNV analysis.

Number of cervical vertebrae	Sum	Percentage (%)	Male	Percentage (%)	Female	Percentage (%)
14	27	21.60	2	7.41	25	92.59
14 (transition state)	27	21.60	11	40.74	16	59.26
15	29	23.20	19	65.52	10	34.48
15 (transition state)	17	13.60	14	82.35	3	17.65
16	25	20.00	23	92.00	2	8.00
Sum	125	100.00	69	55.20	56	44.80

Besides the variations in the number of vertebrae, there existed a great variation in the morphological characteristics of the first thoracic vertebrae (Figure 1B). As an example, the ribs attached to the first thoracic vertebrae showed great difference between ducks with the same phenotype of C15T10 (Figures 1A, B), which indicates a transition of the last cervical vertebrae to the first thoracic vertebrae (Figure 1C).

### Traits’ correlation analysis

To investigate the effect of the number of vertebrae on the economical traits in ducks, the neck and breast productions of ducks with different numbers of vertebrae were compared. As shown in Figure 2B, the number of cervical vertebrae had a significant positive effect on neck weight, neck length, and neck weight percentage. One cervical vertebra addition could increase 11 g or 2 cm of neck on average.

According to the mapping rate of the 352 F2 ducks and 125 Pekin ducks on chromosome W, we identified the sex of all these ducks. In the Pekin ducks × mallard F2 population, the six C16 ducks (ducks with 16 cervical vertebrae) included five male ducks and one female duck, and all the C14 ducks (ducks with 14 cervical vertebrae) were female ducks (Table 1). Among the 125 Pekin ducks with different numbers of cervical vertebrae, 93% of the C14 ducks were female ducks, while 92% of the C16 ducks were male ducks (Figure 2C; Table 3). These results indicated that the male ducks tended to have a greater number of cervical vertebrae. However, the distribution of T9 (ducks with 9 thoracic vertebrae) and T10 ducks (ducks with 10 thoracic vertebrae) did not show any significant difference between male and female ducks (Table 1).

### Copy number variation segments and genotyping

We detected 9,430 unique CNVRs across the 125 Pekin ducks (average length = 4.67 kb) based on the sequence of the read depth against the duck reference genome *duckbase.refseq.v4* (https://www.ncbi.nlm.nih.gov/assembly/GCA_900411745.1), amounting to 44 Mb of the variable sequence or 4% of the duck genome (Supplementary Table S2). Among these detected CNVRs, 1,799 CNVRs were located on an uncharacterized chromosome (chrUn). To filter possibly false CNVs, the CNVs with a minor allele frequency <0.05 were filtered out. Finally, a total of 6,907 CNVRs with high confidence on 29 autosomes, ranging from 4.0 to 269.5 kb, were retained for further analysis (Supplementary Table S2).

A total of 18,238 CNVRs were detected in the Pekin duck × mallard F2 population, among which 2,903 CNVRs were located on unknown chromosomes, 603 were located on the Z chromosome, and 3 were located on the W chromosome. The average length of 18,238 CNVRs was 6.4 kb, the minimum length was 2 kb, and the maximum length was 2,252 kb (Supplementary Table S3). After filtering out CNVRs with boundary factors less than 0.5 and lengths less than 4 kb, there were 1,065 autosomal CNVRs left in the Pekin duck × mallard F2 population with an average length of 17.4 kb (Supplementary Table S3).

### Genome-wide copy number variation association analyses of the number of cervical and thoracic vertebrae

**FIGURE 3:**
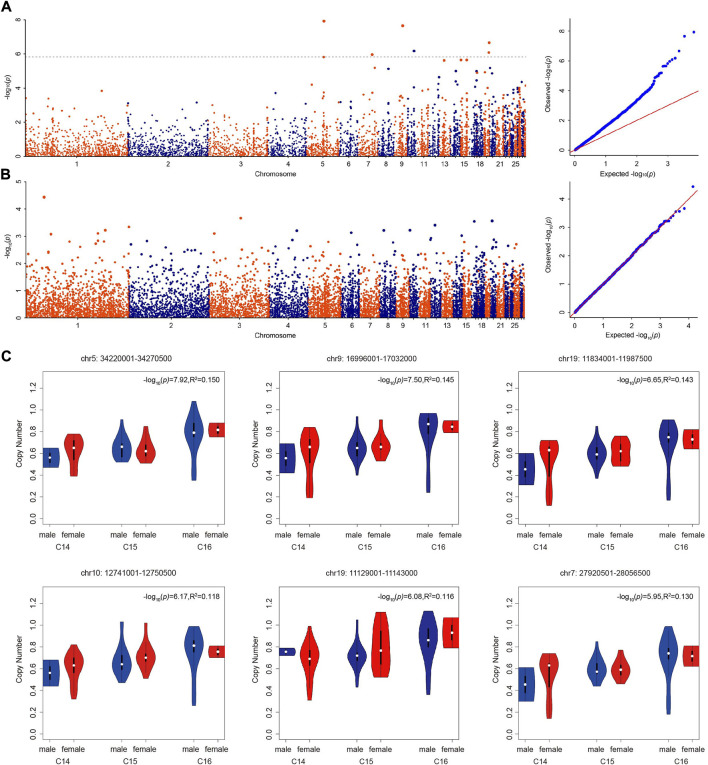
Genome-wide association analyses of the number of vertebrae. **(A)** Manhattan and QQ plots showing the significance of CNV effects on the number of cervical vertebrae. The black dashed line indicates the genome-wide significance of suggestive association (−log_10_(*p*) = 5.84). **(B)** Manhattan and QQ plots showing the significance of CNV effects on the number of thoracic vertebrae. **(C)** The copy number of significant CNVRs (copy number variable regions) in male and female ducks with different numbers of cervical vertebrae.

With the Bonferroni algorithm, 5.84 was used as a significant threshold for a *p*-value of 0.01. The genome-wide association analysis identified six CNVRs associated with the number of cervical vertebrae detected in the genome-wide range located on chromosomes 5, 7, 9, 10, and 19 (Figure 3A; Table 4). All these CNVRs were detected using lumpy-sv as well (Supplementary Table S4). As shown in Supplementary Table S4, the 6 CNVRs overlapped with 22 genes, of which 15 genes have been clearly annotated, which include *VPS37C*, *CD5*, *DES*, *DNPEP*, *WNT10A*, *WNT6*, *POLR2H*, *EPHB3*, *TSC22D3*, *BAIAP2*, *TIMP2*, *CANT1*, *C1QTNF1*, *ENGASE*, and *RBFOX3*. Forty-eight quantitative PCRs were performed to validate the six CNVRs in eight samples. As shown in Supplementary Table S4, over 80% of the results were consistent with the predicted genotypes using CNV caller (Supplementary Table S5).

**TABLE 4:** Basic information of the CNVRs associated with the cervical vertebrae number in the Pekin duck population.

CNV id	Chr	Location	F value	*p* value	R^2^	Add_F value	Add_*p* value	Dom_F value	Dom_*p* value
CNV2620	5	34,220,001–34,270,500	37.41	1.19E-08	0.1504	37.41	1.19E-08	-	-
CNV3524	9	16,996,001–17,032,000	35.81	2.24E-08	0.1454	35.81	2.24E-08	-	-
CNV5369	19	11,834,001–11,987,500	17.43	2.23E-07	0.1433	24.93	2.00E-06	8.41	0.0044
CNV3743	10	12,741,001–12,750,500	27.49	6.74E-07	0.1178	27.49	6.74E-07	-	-
CNV5345	19	11,129,001–11,143,000	26.99	8.30E-07	0.1161	26.99	8.30E-07	-	-
CNV3093	7	27,920,501–28,056,500	15.39	1.10E-06	0.13	20.36	1.49E-05	9.08	0.0031

Using the genome-wide CNV information detected by CNV caller in the Pekin duck × mallard duck F2 population, we conducted a genome-wide association analysis on the number of thoracic vertebrae. The GWAS analysis results are shown in Figure 3B. Using the Bonferroni algorithm, 4.49 was used as the significant threshold for a *p*-value of 0.05. Across the whole genome, no CNVR was significantly associated with the number of thoracic vertebrae.

**FIGURE 4:**
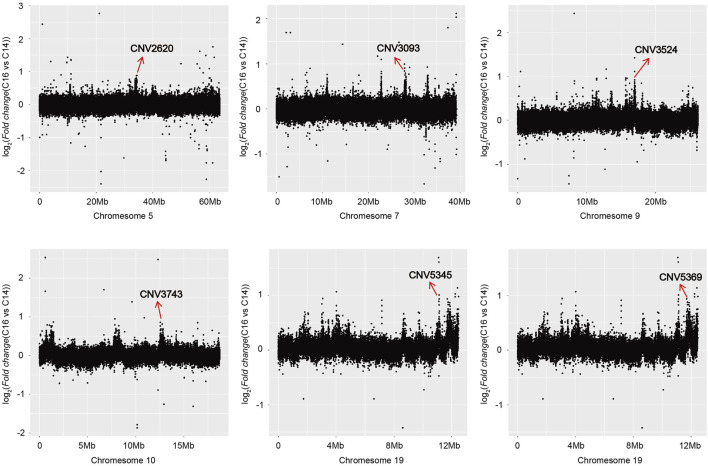
The regions where the absolute copy number shows differences between C14 and C16 in the Pekin duck population in chromosomes 5, 7, 9, 10, and 19.

The copy number of the six associated CNVRs in C14, C15, and C16 populations all showed significant differences which were not influenced by sex (Figure 3C). Using the absolute copy number of each individual in each window obtained by CNVcaller, the ratios of the average copy numbers of the 14 cervical vertebrae and 16 cervical vertebrae groups in each window were calculated. As shown in Figure 4, within the six associated CNV regions detected by GWAS, the average copy number of the 14 cervical vertebrae and 16 cervical vertebrae groups showed remarkable differences. Besides the six CNV regions, there existed other regions that showed absolute copy number differences between the C14 and C16 populations. However, according to the analysis, these CNVRs were mainly associated with sex, and the correlation between these CNVRs and the number of cervical vertebrae disappeared after the addition of the C15 population.

## Discussion

### Variation in the number of vertebrae in duck populations

The number of cervical vertebrae in the Pekin duck population and the Pekin duck × mallard F2 population varied between 14 and 16. As early as 1960, Darwin had found the variation in the number of cervical vertebrae in the mallard population but only reported ducks with 14 and 15 cervical vertebrae (Darwin, 1868). In this study, ducks with 14 and 15 cervical vertebrae were also observed in the duck populations. Furthermore, in Pekin duck and Pekin duck × mallard F2 duck populations, ducks with 16 cervical vertebrae were observed, while in all the populations, most ducks had 15 cervical vertebrae and few ducks had 14 and 16 cervical vertebrae. In a previous study, we investigated variations in the number of cervical vertebrae in Pekin and mallard duck populations (Xu et al., 2019). Compared with the mallard population, the percentage of ducks with 14 cervical vertebrae in the Pekin duck population was lower and the individuals with 16 cervical vertebrae were newly noticed. This indicated that, with artificial breeding, the number of cervical vertebrae has also been slightly selected in the evolution from the mallard to the Pekin duck. In the Pekin duck population from the Z2 line previously reported, the percentage of individuals with 14, 15, and 16 cervical vertebrae were 0%, 94.37%, and 5.63%, respectively (Xu et al., 2019). While in the Pekin duck population from Sai Fei Ya Company, the percentage of ducks with 14, 15, and 16 cervical vertebrae were 5.05%, 94.12%, and 0.82%, respectively. This indicates that the variations in the number of cervical vertebrae differed in different Pekin duck populations. Furthermore, it was observed in multiple populations that male ducks preferred to have more cervical vertebrae when compared to female ducks, which indicated the sex effect on the number of cervical vertebrae.

The variations in the number of thoracic vertebrae was also observed in duck populations and exited association with the number of cervical vertebrae. The ducks with 14 cervical vertebrae always had 10 thoracic vertebrae, and the ones with 16 cervical vertebrae always had 9 thoracic vertebrae. The ducks with 15 cervical vertebrae had 9 or 10 thoracic vertebrae. As with the cervical vertebrae, the variations in the number of thoracic vertebrae differed in different duck populations.

### Influence of the number of vertebrae on ducks’ economic traits

Previous studies on pigs and sheep have indicated that the number of thoracolumbar vertebrae was positively correlated with body length and meat production, and the increase in the number of thoracolumbar vertebrae could raise livestock meat production (Huang et al., 2017; Zhang et al., 2017). In this study, we detected the association between the number of cervical vertebrae and duck neck yield using the data from 421 F2 ducks. Neck weight, length, and neck weight percentage all showed significant differences between ducks with different number of cervical vertebrae. The ducks with more cervical vertebrae had longer and heavier necks. These results are consistent with the conclusions in our previous study, where we also detected high phenotypic and genetic correlations between the number of cervical vertebrae and neck yield (Xu et al., 2019).

### Copy number variation regions' distribution in duck populations

Copy number variations, which widely exist in the livestock and poultry genomes, is an important type of variation in the evolution of animals. The method of mining whole-genome CNV based on resequencing data could be divided into various methods according to the principle. Here, we used CNVcaller based on read depth and lumpy-sv based on sequence pair and split read to analyze high-throughput sequencing data for CNV detection and genotyping (Layer et al., 2014; Wang et al., 2017). While CNV detection using resequencing data usually has certain requirements for sequencing depth, especially the copy number detection method based on read depth, the CNVcaller generally requires a sequencing depth of more than 5× (Wang et al., 2017), and the sequencing depths of the two populations in this study were 5× and 6×, respectively, so the filtering standard for the copy number variation results was required to be higher. Furthermore, the CNVs with greater regions tend to get higher accuracy (Francia et al., 2015).

Using the whole-sequence data, we detected 9,430 CNVRs in the Pekin duck population with an average length of 4.7 kb, while in the Pekin duck × mallard F2 population, a total of 18,238 CNVRs were detected with an average length of 6.4 kb. The number of CNVRs in the Pekin duck × mallard F2 population was much higher than in the Pekin duck, indicating more abundant variations in the Pekin duck × mallard F2 population. In addition, the average length of CNVRs in the F2 population was higher than in the Pekin duck population, which may be due to the presence of more linkage in the genome of the F2 population than natural populations (Slatkin, 2008).

Using resequencing data to detect whole-genome CNVs has been studied in many species, providing CNV profiles for species. Bickhart et al. (2012) conducted whole-genome copy number detection for six cattle (four breeds) using resequencing data based on read depth and detected a total of 1,265 CNV regions in the whole genome. Yi et al. (2014) used resequencing data of 12 different chicken breeds for CNV detection and detected 8,840 CNV regions on a genome-wide scale, accounting for 9.4% of the chicken genome, ranging in size from 1.1 kb to 268.8 kb, with an average of 11.1 kb. Compared with previous study, we used more individuals for CNV detection in this study. Compared with the copy number variations detected in the 12 chicken breeds, the number of CNVs detected in the Pekin duck population in this study did not detect significantly more variations due to the population size, indicating that the abundance of genetic variation is mainly related to the genetic diversity of the population.

### Candidate genes for the number of cervical vertebrae

Among the 15 annotated genes, the *VPS37C* gene in the CNV2620 region encodes a substance that regulates the vesicle transport process and might be involved in cellular growth and differentiation (Eastman et al., 2005). CNV3093 contains seven genes, which include two WNT family genes, *WNT10A* and *WNT6* that are involved in tumorigenesis and several developmental processes as found in previous reports, such as the regulation of cell fate and patterning during embryogenesis (Clevers, 2006). The CNV3524 region contains two genes, *POLR2H* and *EPHB3*, of which *EPHB3* is an ephrin receptor and ligand that mediates many developmental processes (Risley et al., 2009), while the other genes were not reported to be related to the regulation of spine development. Therefore, in the 22 genes found in the 6 CNV regions, only *WNT10A* and *WNT6* were reported to be involved in the regulation of cell fate and patterning during embryogenesis, which might be the candidate genes for the variations in the number of cervical vertebrae. However, the six CNVRs could only explain a small part of the phenotypic variation. According to the variation pattern of the cervical vertebrae, the number of cervical vertebrae was highly correlated with sex. The main influencing mechanism might exist in sex-determining genes. However, sex has an effect on many phenotypes, which brought difficulties to the study on mechanisms of how sex affects the variations in the number of cervical vertebrae.

No CNVRs were detected to be significantly associated with the number of thoracic vertebrae in this study. According to previous studies on the regulation of the axial skeleton in animals, the posterior end of the axial skeleton (the end near the caudal vertebra) usually has a more complex regulatory mechanism than the anterior end of the axial skeleton (Lefebvre and Bhattaram, 2010). In the GWAS analyses, the traits with simpler genetic regulation were easier in detecting the associated genetic variation, which might account for the result that no CNV associated with the number of thoracic vertebrae was detected.

## Conclusion

The number variations of the cervical vertebrae is a rare finding in duck populations, while the number variations of the thoracic vertebrae is a relatively a high-frequency phenomenon in the duck population. The number of cervical vertebrae has a significant positive effect on the duck neck yield. The number variations of the cervical vertebrae is determined by multiple genes and random environmental effects. Six copy number variants were found in this study that could explain part of the number variations of the cervical vertebrae, and *WNT10A* and *WNT6* might be the candidate genes for the number variations of the cervical vertebrae. This study has laid a foundation for the study on variations in bird vertebrae and a possibility for future duck breeding for high neck production.

## Data Availability

Raw sequencing data that support the findings of this study can be found in the NCBI SRA database under the BioProject (PRJNA890937) with the accession numbers SRR22049000, SRR22048978, SRR22048943, SRR22048998, SRR22049006, SRR22048908, SRR22048960, SRR22048961, SRR22048928, SRR22048933, SRR22048959, SRR22049009, SRR22048942, SRR22048964, SRR22048902, SRR22048971, SRR22048898, SRR22048924, SRR22048920, SRR22048901, SRR22048997, SRR22048993, SRR22049001, SRR22048904, SRR22048969, SRR22048937, SRR22048934, SRR22048999, SRR22048945, SRR22049005, SRR22048917, SRR22048923, SRR22048894, SRR22048985, SRR22048925, SRR22048915, SRR22048955, SRR22048977, SRR22048918, SRR22048995, SRR22048976, SRR22048916, SRR22048958, SRR22048910, SRR22048941, SRR22048981, SRR22048903, SRR22048914, SRR22048889, SRR22048983, SRR22048900, SRR22048986, SRR22048940, SRR22048963, SRR22048973, SRR22048906, SRR22048987, SRR22048909, SRR22048996, SRR22048913, SRR22048989, SRR22048984, SRR22048935, SRR22048930, SRR22048951, SRR22049003, SRR22048990, SRR22048895, SRR22048982, SRR22048922, SRR22048992, SRR22049008, SRR22048926, SRR22048972, SRR22048892, SRR22048897, SRR22048893, SRR22048905, SRR22048954, SRR22049002, SRR22048896, SRR22048948, SRR22048952, SRR22048912, SRR22048975, SRR22048970, SRR22048938, SRR22048932, SRR22048947, SRR22048980, SRR22048949, SRR22048953, SRR22048950, SRR22048931, SRR22048991, SRR22048994, SRR22048907, SRR22048967, SRR22048968, SRR22048946, SRR22049012, SRR22048988, SRR22048974, SRR22048962, SRR22048927, SRR22048957, SRR22048891, SRR22049010, SRR22048939, SRR22048966, SRR22049004, SRR22048890, SRR22048965, SRR22048888, SRR22048956, SRR22048919, SRR22048921, SRR22049007, SRR22048936, SRR22049011, SRR22048911, SRR22048979, SRR22048929, SRR22048899, SRR22048944, and other data was available in NCBI’s Sequence Read Archive database (Accession number: CRA003272).
