# Supplementary material for: Genome-wide association analysis reveals 6 copy number variations associated with the number of cervical vertebrae in Pekin ducks
Source: Front Cell Dev Biol. 2022 Nov 10;10:1041088. doi: 10.3389/fcell.2022.1041088 (PMC9685309; doi:10.3389/fcell.2022.1041088)
Supplement: Supplementary file 1 [file Table1.docx]

**Table S1.** Prinmers information for CNV (copy number variation) validation.

| **CNV** | **Primer sequences（5`-3`）** | **Product size** |
| --- | --- | --- |
| CNV2620 | Forward: GTCAGCTGCCAAAGAAGGAC | 166 |
|  | Reverse: CTAAAGCCCGCTGCAAATAG |  |
| CNV3093 | Forward: CCTTGTGGGACCTTGTCTGT | 167 |
|  | Reverse: CAGAGCCTTGCAAAACCTTC |  |
| CNV3524 | Forward: ACACCGAAATAAAGCCATCG | 218 |
|  | Reverse: AGCCCTCAACCAGGGTAACT |  |
| CNV3743 | Forward: CAACGAAGAGTCTTGCACCA | 206 |
|  | Reverse: CCCTTTCAGAGGGAAAGACC |  |
| CNV5345 | Forward: GCCAACCTGACGATTTTGAT | 211 |
|  | Reverse: GCTCAGCCTTTTCTGACACC |  |
| CNV5369 | Forward: CAAACTGCTCCAGGAAAAGC | 194 |
|  | Reverse: CCGGCTTCTTTCAGTTCTTG |  |
| CNV1332 | Forward: GGTATGCGTGCCTCATTTTT | 186 |
|  | Reverse: AGAAGCAACAAAAGGGCTGA |  |
| *Ldh-B* | Forward: TTAATGTGGCAGGCGTTTCT | 107 |
|  | Reverse: AGGCACTTTCAACCACTTGC |  |
