# Supplementary material for: Genome-wide association analysis reveals 6 copy number variations associated with the number of cervical vertebrae in Pekin ducks
Source: Front Cell Dev Biol. 2022 Nov 10;10:1041088. doi: 10.3389/fcell.2022.1041088 (PMC9685309; doi:10.3389/fcell.2022.1041088)
Supplement: Supplementary file 2 [file Table2.docx]

**Table S2.** The genome-wide CNVRs information in Pekin duck population.

| **Chromosome** | **Before filter** | | | | **After filter** | | | |
| --- | --- | --- | --- | --- | --- | --- | --- | --- |
|  | **N** | **Average length/kb** | **Min**  **/kb** | **Max**  **/kb** | **N** | **Average length/kb** | **Min**  **/kb** | **Max**  **/kb** |
| 1 | 983 | 3.0 | 2.0 | 37.0 | 61 | 8.9 | 4.0 | 37.0 |
| 2 | 709 | 3.1 | 2.0 | 55.5 | 52 | 8.5 | 4.0 | 55.5 |
| 3 | 486 | 3.3 | 2.0 | 47.0 | 38 | 9.4 | 4.0 | 47.0 |
| 4 | 322 | 2.9 | 2.0 | 25.0 | 14 | 8.7 | 4.0 | 25.0 |
| 5 | 382 | 3.4 | 2.0 | 61.0 | 29 | 8.3 | 4.0 | 50.5 |
| 6 | 196 | 3.9 | 2.0 | 114.5 | 17 | 12.8 | 4.0 | 114.5 |
| 7 | 203 | 3.9 | 2.0 | 136.0 | 11 | 7.7 | 4.0 | 14.5 |
| 8 | 232 | 4.6 | 2.0 | 269.5 | 17 | 23.5 | 4.0 | 269.5 |
| 9 | 203 | 3.9 | 2.0 | 39.5 | 16 | 9.6 | 4.0 | 39.5 |
| 10 | 202 | 3.6 | 2.0 | 14.0 | 26 | 6.6 | 4.0 | 13.0 |
| 11 | 204 | 3.9 | 2.0 | 80.0 | 16 | 7.1 | 4.0 | 29.0 |
| 12 | 178 | 4.2 | 2.0 | 54.5 | 12 | 15.2 | 4.0 | 54.5 |
| 13 | 180 | 4.0 | 2.0 | 35.0 | 21 | 10.6 | 4.0 | 35.0 |
| 14 | 232 | 3.7 | 2.0 | 24.0 | 20 | 8.8 | 4.0 | 24.0 |
| 15 | 227 | 4.2 | 2.0 | 36.5 | 31 | 8.7 | 4.0 | 36.5 |
| 16 | 192 | 3.8 | 2.0 | 40.0 | 22 | 5.8 | 4.0 | 19.0 |
| 17 | 20 | 5.2 | 2.0 | 25.0 | 2 | 14.5 | 4.0 | 25.0 |
| 18 | 208 | 3.5 | 2.0 | 45.0 | 14 | 6.6 | 4.0 | 17.5 |
| 19 | 199 | 4.7 | 2.0 | 153.5 | 23 | 6.9 | 4.0 | 19.0 |
| 20 | 189 | 4.0 | 2.0 | 38.0 | 17 | 8.7 | 4.0 | 30.0 |
| 21 | 188 | 3.7 | 2.0 | 31.0 | 16 | 5.8 | 4.0 | 10.0 |
| 22 | 117 | 3.8 | 2.0 | 41.5 | 9 | 6.8 | 4.5 | 16.0 |
| 23 | 71 | 3.7 | 2.0 | 17.5 | 4 | 7.1 | 4.0 | 16.5 |
| 24 | 156 | 4.6 | 2.0 | 74.0 | 19 | 7.9 | 4.0 | 18.5 |
| 25 | 113 | 3.5 | 2.0 | 31.0 | 11 | 6.0 | 4.0 | 9.5 |
| 26 | 65 | 5.0 | 2.0 | 27.0 | 10 | 7.5 | 4.0 | 15.0 |
| 27 | 235 | 3.8 | 2.0 | 17.0 | 35 | 6.9 | 4.0 | 17.0 |
| 28 | 118 | 4.6 | 2.0 | 35.5 | 17 | 7.8 | 4.0 | 22.0 |
| 29 | 97 | 4.6 | 2.0 | 21.0 | 9 | 8.2 | 4.0 | 21.0 |
| Z | 399 | 6.6 | 2.0 | 1870.0 | 0 | - | - | - |
| W | 1 | 1087.5 | 1087.5 | 1087.5 | 0 | - | - | - |
| U | 2123 | 9.6 | 2 | 264.5 | 0 | - | - | - |
| SUM | 9430 | 4.7 | 2.0 | 1870.0 | 589 | 8.8 | 4.0 | 269.5 |
