# Supplementary material for: Genome-wide association analysis reveals 6 copy number variations associated with the number of cervical vertebrae in Pekin ducks
Source: Front Cell Dev Biol. 2022 Nov 10;10:1041088. doi: 10.3389/fcell.2022.1041088 (PMC9685309; doi:10.3389/fcell.2022.1041088)
Supplement: Supplementary file 3 [file Table5.docx]

**Table S5.** CNV validation on the 6 associated CNVR with qPCR.

| **Sample name** | **CNV2620** | | **CNV3093** | | **CNV3524** | | **CNV3743** | | **CNV5345** | | **CNV5369** | |
| --- | --- | --- | --- | --- | --- | --- | --- | --- | --- | --- | --- | --- |
|  | **2^-ΔΔCT^** | **CN** | **2^-ΔΔCT^** | **CN** | **2^-ΔΔCT^** | **CN** | **2^-ΔΔCT^** | **CN** | **2^-ΔΔCT^** | **CN** | **2^-ΔΔCT^** | **CN** |
| sample 1 | 1.61 | 0.5 | 0.87 | 0.5 | 0.51 | 0.5 | 0.77 | 0.5 | 1.2 | 1 | 0.99 | 0.5 |
| sample 2 | 1.27 | 1 | 3.63 | 1 | 1.68 | 1 | 1.87 | 1 | 1.56 | 1 | 0.92 | 1 |
| sample 3 | 0.67 | 1 | 1.7 | 1 | 3.26 | 1 | 0.43 | 0.5 | 0.47 | 0.5 | 0.85 | 0.5 |
| sample 4 | 2.39 | 1 | 0.19 | 0.5 | 0.36 | 0.5 | 1.62 | 1 | 1.13 | 0.5 | 1.29 | 1 |
| sample 5 | 0.34 | 0.5 | 0.13 | 0.5 | 0.54 | 0.5 | 7.38 | 1 | 0.98 | 1 | 1.06 | 0.5 |
| sample 6 | 6.64 | 1 | 0.19 | 0.5 | 0.37 | 1 | 0.62 | 0.5 | 0.59 | 0.5 | 0.84 | 0.5 |
| sample 7 | 0.47 | 0.5 | 0.09 | 0.5 | 0.39 | 0.5 | 0.58 | 1 | 0.8 | 1 | 1.02 | 1 |
| sample 8 | 0.29 | 0.5 | 0.21 | 0.5 | 0.59 | 1 | 0.45 | 0.5 | 0.88 | 0.5 | 1.58 | 0.5 |
