# Supplementary material for: Genome-wide association analysis reveals 6 copy number variations associated with the number of cervical vertebrae in Pekin ducks
Source: Front Cell Dev Biol. 2022 Nov 10;10:1041088. doi: 10.3389/fcell.2022.1041088 (PMC9685309; doi:10.3389/fcell.2022.1041088)
Supplement: Supplementary file 4 [file Table3.docx]

**Table S3.** The genome-wide CNVRs information in Pekin duck × mallard F2 population.

| **Chromosome** | **Before filter** | | | | **After filter** | | | |
| --- | --- | --- | --- | --- | --- | --- | --- | --- |
|  | **N** | **Average length/kb** | **Min**  **/kb** | **Max**  **/kb** | **N** | **Average length/kb** | **Min**  **/kb** | **Max**  **/kb** |
| 1 | 2324 | 4.2 | 2.0 | 436.0 | 293 | 11.8 | 4.0 | 436.0 |
| 2 | 1590 | 4.1 | 2.0 | 330.5 | 188 | 11.7 | 4.0 | 330.5 |
| 3 | 1131 | 4.2 | 2.0 | 239.5 | 125 | 15.1 | 4.0 | 239.5 |
| 4 | 803 | 3.6 | 2.0 | 116.5 | 70 | 9.4 | 4.0 | 116.5 |
| 5 | 935 | 4.3 | 2.0 | 369.5 | 106 | 14.5 | 4.0 | 369.5 |
| 6 | 742 | 6.7 | 2.0 | 1011.5 | 133 | 19.5 | 4.0 | 1011.5 |
| 7 | 470 | 4.2 | 2.0 | 174.5 | 49 | 13.6 | 4.0 | 174.5 |
| 8 | 580 | 4.6 | 2.0 | 269.5 | 83 | 13.1 | 4.0 | 269.5 |
| 9 | 434 | 4.7 | 2.0 | 205.0 | 61 | 13.2 | 4.0 | 205.0 |
| 10 | 386 | 6.6 | 2.0 | 342.5 | 59 | 11.1 | 4.0 | 95.5 |
| 11 | 405 | 5.7 | 2.0 | 451.0 | 47 | 21.2 | 4.0 | 451.0 |
| 12 | 343 | 6.1 | 2.0 | 446.5 | 47 | 13.2 | 4.0 | 79.5 |
| 13 | 350 | 4.8 | 2.0 | 114.0 | 48 | 11.4 | 4.0 | 47.0 |
| 14 | 453 | 6.8 | 2.0 | 534.5 | 71 | 23.7 | 4.0 | 534.5 |
| 15 | 473 | 5.8 | 2.0 | 271.0 | 77 | 13.3 | 4.0 | 271.0 |
| 16 | 329 | 12.0 | 2.0 | 2252.0 | 56 | 52.3 | 4.0 | 2252.0 |
| 17 | 27 | 11.0 | 2.0 | 34.0 | 10 | 16.1 | 4.0 | 34.0 |
| 18 | 421 | 4.9 | 2.0 | 219.5 | 59 | 8.4 | 4.0 | 73.0 |
| 19 | 327 | 11.3 | 2.0 | 1731.5 | 55 | 12.5 | 4.0 | 62.0 |
| 20 | 349 | 6.2 | 2.0 | 268.5 | 48 | 21.7 | 4.0 | 268.5 |
| 21 | 384 | 5.1 | 2.0 | 109.5 | 53 | 11.2 | 4.0 | 86.0 |
| 22 | 268 | 6.5 | 2.0 | 444.0 | 36 | 10.8 | 4.0 | 52.0 |
| 23 | 33 | 82.3 | 2.0 | 2018.0 | 8 | 78.3 | 4.0 | 473.5 |
| 24 | 282 | 9.2 | 2.0 | 750.5 | 47 | 33.3 | 4.0 | 750.5 |
| 25 | 279 | 4.2 | 2.0 | 132.5 | 35 | 7.8 | 4.0 | 30.0 |
| 26 | 9 | 140.1 | 2.5 | 847.0 | 6 | 151.1 | 4.0 | 847.0 |
| 27 | 210 | 20.0 | 2.0 | 1201.0 | 31 | 56.5 | 4.0 | 762.0 |
| 28 | 244 | 8.7 | 2.0 | 776.0 | 34 | 15.8 | 4.0 | 144.0 |
| 29 | 148 | 16.2 | 2.0 | 770.5 | 30 | 61.6 | 4.0 | 770.5 |
| Z | 603 | 7.5 | 2.0 | 1870.0 | 0 | - | - | - |
| W | 3 | 358.3 | 2.0 | 669.0 | 0 | - | - | - |
| U | 2903 | 8.6 | 2.0 | 679.0 | 0 | - | - | - |
| SUM | 18238 | 6.4 | 2.0 | 2252.0 | 1965 | 17.4 | 4.0 | 2252.0 |
