# Supplementary material for: Genome-wide association analysis reveals 6 copy number variations associated with the number of cervical vertebrae in Pekin ducks
Source: Front Cell Dev Biol. 2022 Nov 10;10:1041088. doi: 10.3389/fcell.2022.1041088 (PMC9685309; doi:10.3389/fcell.2022.1041088)
Supplement: Supplementary file 5 [file Table4.docx]

**Table S4.** Genes distributed in the significant associated CNVRs.

| **CNV** | **Chromosome** | **CNV caller** | | **Lumpy-sv** | | **P-value** | **genes** |
| --- | --- | --- | --- | --- | --- | --- | --- |
|  |  | **from** | **to** | **from** | **to** |  |  |
| CNV2620 | 5 | 34,220,001 | 34,270,500 | 34,222,785 | 34,270,326 | 0.000082 | *VPS37C* |
|  |  |  |  |  |  |  | *CD5* |
|  |  |  |  |  |  |  | *LOC101795164* |
|  |  |  |  |  |  |  | *LOC101801089* |
|  |  |  |  |  |  |  | *LOC101795359* |
| CNV3093 | 7 | 27,920,501 | 28,056,500 | 27,924,546 | 28,056,091 | 0.000155 | *LOC106017566* |
|  |  |  |  |  |  |  | *LOC106017565* |
|  |  |  |  |  |  |  | *DES* |
|  |  |  |  |  |  |  | *DNPEP* |
|  |  |  |  |  |  |  | *WNT10A* |
|  |  |  |  |  |  |  | *WNT6* |
| CNV3524 | 9 | 16,996,001 | 17,032,000 | 16,996,488 | 17,031,237 | 0.001539 | *LOC101802865* |
|  |  |  |  |  |  |  | *POLR2H* |
|  |  |  |  |  |  |  | *EPHB3* |
| CNV3743 | 10 | 11,834,001 | 11,987,500 | 12,743,516 | 12,750,161 | 0.004653 | *TSC22D3* |
| CNV5345 | 19 | 12,741,001 | 12,750,500 | 11,129,959 | 11,141,701 | 0.005729 | *BAIAP2* |
| CNV5369 | 19 | 11,129,001 | 11,143,000 | 11,834,143 | 11,986,148 | 0.007593 | *TIMP2* |
|  |  |  |  |  |  |  | *CANT1* |
|  |  |  |  |  |  |  | *C1QTNF1* |
|  |  |  |  |  |  |  | *ENGASE* |
|  |  |  |  |  |  |  | *RBFOX3* |
|  |  |  |  |  |  |  | *LOC106014562* |
