# Supplementary figures and images for: Genome-wide association analysis reveals 6 copy number variations associated with the number of cervical vertebrae in Pekin ducks
Source: Front Cell Dev Biol. 2022 Nov 10;10:1041088. doi: 10.3389/fcell.2022.1041088 (PMC9685309; doi:10.3389/fcell.2022.1041088)

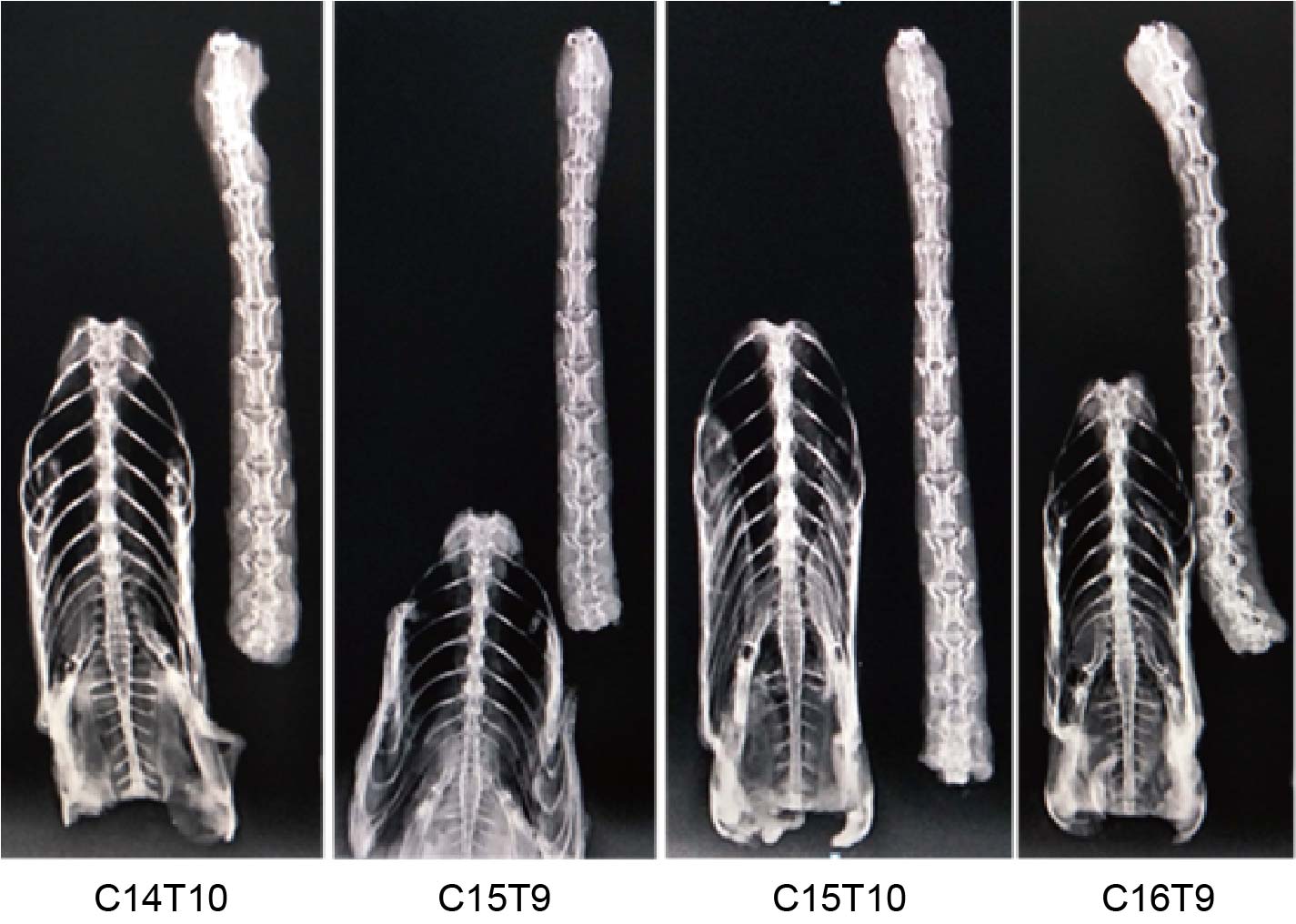

Supplement: Supplementary file 6 [file Image1.jpg]
